# Evaluation of Lipid Changes During the Drying Process of *Cordyceps sinensis* by Ultra Performance Liquid Chromatography–Tandem Mass Spectrometry (UPLC-MS/MS)-Based Lipidomics Technique

**DOI:** 10.3390/jof10120855

**Published:** 2024-12-11

**Authors:** Mengjun Xiao, Tao Wang, Chuyu Tang, Min He, Yuling Li, Xiuzhang Li

**Affiliations:** State Key Laboratory of Plateau Ecology and Agriculture, Qinghai Academy of Animal and Veterinary Science, Qinghai University, Xining 810016, China; 15574237597@163.com (M.X.); 13085500761@163.com (T.W.); chuyutang0410@163.com (C.T.); himi1228@163.com (M.H.); yulingli2000@163.com (Y.L.)

**Keywords:** *Cordyceps sinensis*, drying methods, multiple reaction monitoring (MRM), lipidomic, oxidation

## Abstract

Comprehensive analysis of the lipid content in *Cordyceps sinensis* samples is essential for optimizing their effective use. Understanding the lipid profile can significantly enhance the application of this valuable fungus across various fields, including nutrition and medicine. However, to date, there is limited knowledge regarding the effects of different drying methods on the quality of lipids present in *Cordyceps sinensis*. In this study, we employed a broadly targeted lipidomic strategy to conduct a comprehensive analysis of the lipid composition in *Cordyceps sinensis* subjected to various drying methods. A comprehensive analysis identified a total of 765 distinct lipid species from fresh *Cordyceps sinensis* (FC), vacuum-freeze-dried *Cordyceps sinensis* (VG), oven-dried *Cordyceps sinensis* (OG), and air-dried *Cordyceps sinensis* (AG). Among these, glycerophospholipids (GP) were the most abundant, followed by glycerides (GL) and sphingolipids (SP). In this study, a total of 659 lipids demonstrated statistically significant differences, as indicated by a *p*-value (*p*) < 1. Among these lipids, triglycerides (TG) exhibited the highest concentration, followed by several others, including ceramide-ascorbic acid (Cer-AS), phosphatidylethanolamine (PE), lysophosphatidylcholine (LPC), and phosphatidylserine (PS). OG was the fastest drying method; however, PCA and OPLS-DA analyses indicated that the most significant changes in the lipids of *Cordyceps sinensis* were observed under the OG method. Specifically, 517 differentially accumulated lipids were significantly down-regulated, while only 10 lipids were significantly up-regulated. This disparity may be attributed to the degradation and oxidation of lipids. The metabolic pathways of glycerolipid, glycerophospholipid, and cholesterol are critical during the drying process of *Cordyceps sinensis*. This study provides valuable insights that can enhance quality control and offer guidelines for the appropriate storage of this medicinal fungus.

## 1. Introduction

*Cordyceps sinensis* is a valuable fungus with a variety of biological and pharmacological functions and a rich history of medicinal applications and is widely used for promoting human health and preventing diseases [1,2]. The natural resources of *Cordyceps sinensis* are scarce due to its harsh growing conditions, limited geographic distribution, and the impacts of global warming, with the fungus primarily found in alpine regions at altitudes of 3000 to 5000 m [3,4]. Numerous studies have confirmed that *Cordyceps sinensis* has a range of pharmacological effects including significantly reducing inflammatory responses, enhancing immune responses, combating oxidative stress, and anticancer properties. The potential of *C. sinensis* as a therapeutic agent in various medical applications is noteworthy, highlighting the necessity for further investigation into its benefits and mechanisms of action [5,6,7]. In recent years, researchers have isolated and characterized a variety of compounds from *C. sinensis*, discovering active ingredients with diverse pharmacological effects that are widely utilized in clinical treatment [8,9]. 

*C. sinensis* is highly valued for its significant lipid constituents and high abundance of unsaturated fatty acids [10,11]. It is regarded as a valuable source of lipids due to its rich content of phospholipids (PLs) and fatty acids (FFAs), both of which provide notable health benefits [12,13]. The substance is rich in essential components, including minerals, vitamins, and amino acids [14,15]. Renowned globally as the “king of all medicines”, it is recognized as a nourishing food that may provide lifespan-enhancing benefits and can be consumed in its entirety [16]. However, like other fungal substances, *C. sinensis* is susceptible to degradation due to its high water content and elevated levels of active enzymes [17,18]. Therefore, it is recommended to refrigerate or freeze promptly to maintain its freshness and quality [19,20]. Although effective refrigeration and freezing methods can extend the shelf life of *C. sinensis*, ensuring quality over an extended period can be challenging and costly.

Among various preservation techniques, drying effectively prolongs the shelf life of *C. sinensis* by inhibiting microbial growth, reducing enzymatic activity, and slowing chemical reactions, all while minimizing quality deterioration [21,22,23]. The market value of the product is further enhanced by drying, which reduces costs associated with packaging, storage, and transportation. As a result, several drying techniques, including oven drying (OG) [24], vacuum freeze-drying (VG) [25], and air drying (AG) [26], have been developed and employed for fungus-based products. Among the various methods available, OG stands out as the most widely utilized technique within the fungus-based processing sector due to its rapid processing duration and ease of management, which together enhance the shelf life and preserve the quality of the products. However, in comparison to alternative preservation techniques, OG leads to the degradation of several heat-sensitive compounds found in *C. sinensis*, such as lipids and proteins [27,28]. Several factors contribute to the breakdown of lipids during the oven drying process, including exposure to high temperatures, the duration of drying, and oxidative reactions [29,30].

Lipids play a crucial role in numerous cellular processes, primarily serving structural and energy storage functions, and understanding the lipid composition and metabolism of *C. sinensis* is essential for elucidating its biological functions and interactions with hosts and microbes. Consequently, this study focused on various bioactive lipids found in DALs, particularly fatty acids (such as unsaturated free fatty acids and eicosanoids) and glycerophospholipids (including phosphatidylglycerol, phosphatidylcholine, phosphatidylethanolamine, and phosphatidylinositol). Free fatty acids (FFAs) are an important source of energy for human tissues and possess physiological functions, including the promotion of insulin secretion and anti-inflammatory effects [31]. Eicosanoids are believed to mediate their signaling effects only in the form of free acids, which can regulate renal, cardiovascular, and gastrointestinal functions. Diacylglycerols (DGs) are glycerides, a class of esters produced by the esterification of glycerol and fatty acids [32]. PG, PC, PE, and PI, as the four major GPs, play significant roles in promoting cell and tissue growth, maintaining metabolism, and enhancing the body’s immunity and regenerative capacity [33]. Due to the complex nature and importance of lipid metabolism, as well as the potential applications of lipids, a burgeoning field known as lipidomics—considered a sub-discipline of metabolomics—has emerged. Recent advancements in mass spectrometry (MS) technology [34,35] have led to significant improvements in its applications, sensitivity, and reliability. Furthermore, lipidomic analysis has been conducted on various medicinal fungi, with related studies employing lipidomics to elucidate the dynamic lipid changes occurring at different growth stages of *Flammulina velutipes*. The results of this research have greatly elucidated the lipid profiles associated with the morphology and growth of the alga *Flammulina velutipes*, thus laying the groundwork for future research in agricultural and food chemistry applications and contributing to the improvement of the industrial production and quality management of filaments [36]. Additionally, a separate study utilized lipidomics to analyze changes in lipid metabolites in rats suffering from hyperlipidemia induced by a high-fat diet, as well as to investigate the effectiveness and underlying mechanisms of *Flammulina velutipes* in mitigating diet-induced hyperlipidemia [37].

At present, there are limited studies on the effects of various drying methods on the lipids of *C. sinensis*. In this work, liquid chromatography–tandem mass spectrometry (LC-MS/MS) was used to analyze the lipid composition, content, and key metabolic pathways of fresh *C*. *sinensis* treated with vacuum freeze-drying (VG), oven drying (OG), and air drying (AG).

## 2. Materials and Methods

### 2.1. Materials and Chemicals

*C. sinensis* (FC) was supplied by Qinghai Baohuitang Biotechnology Co. (Qinghai, China) and sourced from Maqin County (94°35′ E, 34°08′ N) in the Guoluo Tibetan Autonomous Prefecture of Qinghai Province, China, with a total weight of 20 g. Only well-preserved and undeteriorated specimens were selected for the experiment, and they were stored at −80 °C. Methanol, acetonitrile, isopropyl alcohol, ammonium formate, and methyl tert-butyl ether were acquired from Merck Drugs & Biotechnology Co., Ltd., Darmstadt, Germany. Formic acid, a key reagent often used in chemical synthesis and chromatography, was sourced from Sigma-Aldrich Trading Co., Ltd., Shanghai, China. Ammonium formate and dichloromethane, which are commonly used in various applications, were purchased from Thermo Fisher Scientific Co., Ltd., Shanghai, China. To standardization, specific compounds including 12:0 Lyso PC,Cer(d18:1/4:0), and PC(13:0/13:0) were obtained from zzstandard Technology Co., Ltd., Shanghai, China, ensuring the availability of high-quality standards necessary for the accurate assessment of analytical results.

### 2.2. Sample Preparation and Lipid Extraction

Vacuum freeze-drying (VG) is a method in which fresh *C. sinensis* (FC) is pre-cooled at −35 °C for 3 h, followed by 25 h of low-temperature, high-vacuum freeze-drying chamber (Pilot 5-8S, Blocool Experimental Instruments Co., Ltd., Beijing, China) to ensure that the samples achieve a constant weight. The oven-drying method (OG) involved placing fresh *C. sinensis* (FC) in an electric thermostatic drying oven (PH030A, Yiheng Technology Co., Ltd., Shanghai, China). In this method, the samples were uniformly spread across the tray and subjected to hot air at a temperature of 60 °C until they also reached a stable weight, after which the dried samples were removed from the oven. The shade drying method involved laying the fresh *C. sinensis* (FC) flat in a cool, dry location within an air-conditioned environment. The ambient conditions maintained during the drying process ranged from 20 to 25 °C, with a relative humidity of 50% to 65%. These conditions allowed the samples to achieve a constant weight naturally over time. For the lipidomic analysis, three biological replicates were prepared for each drying method, resulting in a total of twelve sample groups. The samples were mixed thoroughly to produce quality control (QC) samples, which were essential for monitoring the equilibrium and stability of the UPLC-MS/MS system. Finally, all prepared samples were stored at −80 °C to ensure their preservation for subsequent analyses.

Lipid extraction was conducted using the methods outlined in the related studies and adapted from existing laboratory protocols [38,39]. Following thawing, precisely 20 mg (±1 mg) of the sample was weighed into each set of distinctly labeled centrifuge tubes. Subsequently, 1 mL of Lipid Extraction Solution (comprising methyl tert-butyl ether and Methanol in a 3:1 volume ratio) containing the internal standard was added (see Table S1 for complete information on internal). The mixture was vortexed for 15 min, after which 200 μL of water was introduced, and the mixture was vortexed for an additional minute. The samples were then centrifuged for 10 min at 12,000 rpm at a temperature of 4 °C. The supernatant was carefully aspirated into the corresponding numbered centrifuge tubes. The samples were concentrated to complete dryness, after which 200 μL of Lipid Replicate Solution (with acetonitrile and isopropanol in a 3:1 volume ratio) was added to each tube. Finally, the supernatant was again concentrated to dryness, and 200 μL of lipid complex solution was added to make the composition of the solvent and mobile phase consistent (comprising acetonitrile and isopropanol in a 1:1 volume ratio), followed by vortexing for 3 min and centrifuging at 12,000 rpm/min for an additional 3 min. The supernatant was then pipetted for subsequent UPLC-MS/MS analysis.

### 2.3. Lipidomics Data Collection Conditions

The data acquisition instrumentation system primarily consists of Ultra Performance Liquid Chromatography (UPLC, ExionLC AD) (Thermo Fisher Scientific Co., Ltd., Shanghai, China) and Tandem Mass Spectrometry (MS/MS, QTRAP^®^-6500+) (Thermo Fisher Scientific Co., Ltd., Shanghai, China). The chromatographic column utilized in the analysis is a Thermo Accucore™ C30 (Thermo Fisher Scientific Co., Ltd., Shanghai, China) column, characterized by a particle size of 2.6 μm and dimensions of 2.1 mm × 100 mm internal diameter. The system operates at a flow rate of 0.35 mL/min, with the column maintained at a temperature of 45 °C. An injection volume of 2 μL is employed for sample introduction into the system. Regarding the mobile phases, the study adopts a dual-phase approach. Phase A consists of a mixture of acetonitrile and water in a 60:40 (*v*/*v*) ratio, further enhanced by the addition of 0.1% formic acid and 10 mmol of ammonium formate. In contrast, Phase B comprises acetonitrile and isopropanol in a 10:90 (*v*/*v*) ratio, which also includes 0.1% formic acid and 10 mmol of ammonium formate. The gradient profile for the mobile phase transitions through various compositions over specific time intervals. Initially, at time zero, the mobile phase is set at a ratio of 80:20 for phases A and B, respectively. This ratio shifts to 70:30 at 2 min, 40:60 at 4 min, and continues to progress to a final ratio of 80:20 by 20 min, thereby facilitating optimal elution of analytes throughout the chromatographic run. For the tandem mass spectrometry analysis, the electrospray ionization (ESI) temperature is established at 500 °C, ensuring efficient ionization of the analytes. The mass spectral voltage is configured at 5500 V in positive ion mode and −4500 V in negative ion mode, which enhances detection sensitivity. The ion source gas pressures are set at 45 psi for gas1 (Nitrogen, Auburn, CA, USA) and 55 psi for gas2 (Nitrogen), with a curtain gas pressure of 35 psi. Additionally, the collision-activated dissociation (CAD) parameter is calibrated to a medium setting, promoting effective fragmentation of ions for subsequent analysis. Each ion pair within the triple quadrupole mass spectrometer is systematically scanned and detected, utilizing optimized declustering potential (DP) and collision energy (CE) parameters that have been fine-tuned to enhance detection quality. Lipid quantification and data analysis are performed accordingly.

### 2.4. Lipidomics Quantification and Data Analysis

Based on previous research [39] and utilizing the self-constructed Metware Database (MWDB), along with public databases such as mzCloud (https://www.mzcloud.org, accessed on 1 September 2024), HMDB (https://www.hmdb.ca, accessed on 1 September 2024), and MassBank (http://www.massbank.jp/, accessed on 1 September 2024), a qualitative analysis was conducted focusing on retention time (RT) and the daughter ion pair information of the detected substances. This analysis employed multiple reaction monitoring (MRM) mode through triple quadrupole mass spectrometry. Within the MRM framework, the four-pole rod initially identifies the precursor ion (parent ion) of the target compound while filtering out ions associated with substances of different molecular weights to minimize interference. Subsequently, the precursor ion is ionized in the collision chamber, yielding numerous fragment ions. These fragment ions are then processed through the triple quadrupole rod to isolate the specific fragment ion required, thereby reducing the influence of non-target ions and enhancing both the accuracy and reproducibility of the quantification.

The raw data, which included cations (POS) and anions (NEG), were pre-processed by initially filling in missing values using one-fifth of the smallest value from each row (lipid). Following this, the coefficient of variation (CV) values of the quality control (QC) samples were calculated, retaining only those with a CV value of less than 0.3 to create the final data file. The mass spectrometry data were analyzed using Analyst 1.6.3 software, where characteristic ions for each substance were identified through a triple quadrupole, and the signal intensity (CPS) was recorded by the detector. The mass spectrometry files for the samples were accessed via MultiQuant software (https://sciex.com/products/software/multiquant-software, accessed on 1 September 2024), which facilitated the integration and calibration of chromatographic peaks. The area of each peak corresponded to the relative concentration of the respective substance, and the final data for all chromatographic peaks were exported to preserve the peak area integration information. To enable a comparison of lipid content across different samples for all detected lipids, we adjusted the chromatographic peaks of each lipid found in various samples. This adjustment was based on lipid retention time and peak shape information, ensuring the precision of both qualitative and quantitative analyses.

### 2.5. Data Stability and Statistical Analysis

Quality control (QC) samples are generated by combining 12 sample extracts, which are employed to evaluate the consistency of samples processed using the same method [40]. The consistency of lipid extraction and detection can be assessed through the overlay analysis of total ion current (TIC) plots obtained from various QC samples analyzed via mass spectrometry. Additionally, overlay plots illustrating the TIC profiles of QC samples examined through mass spectrometry detection are also utilized (Figure S1A,B). The findings revealed a significant overlap in the total ion flow curves for lipid detection, indicating that both the retention time and peak intensity remained consistent. This consistency suggests that mass spectrometry exhibits strong signal stability when analyzing the same sample at different times, with NEG representing the negative ion mode and POS indicating the positive ion mode. Additionally, the coefficient of variation (CV) for the quality control (QC) samples exceeded 85%, demonstrating that the assay and the obtained data are highly reliable (Figure S2).

Lipid data were normalized and log-transformed prior to the execution of multivariate statistical analysis. The statistical function ‘promp’ in R (version 3.5.0) was utilized to conduct principal component analysis (PCA). For orthogonal partial least squares discriminant analysis (OPLS-DA), the R package MetaboAnalyst R (version 1.0.1) was employed. Heatmaps were used to facilitate hierarchical cluster analysis (HCA), illustrating the fluctuations in lipid metabolites during the drying process. To identify differential lipids, the statistical significance projection variable importance threshold (VIP) and *p*-value (*p*) parameters were applied, with a VIP greater than 1 and a *p*-value less than 0.05 regarded as indicative of differential lipids. To mitigate overfitting, permutation tests involving 200 permutations were conducted. Additionally, differential lipid analysis (DALs) was mapped to metabolic pathways for functional classification and pathway enrichment analysis using MetaboAnalyst.ca, version 6.0.

## 3. Results

### 3.1. Statistical Analysis of Lipid Characteristics of C. sinensis Under Different Drying Methods

**Figure 1:**
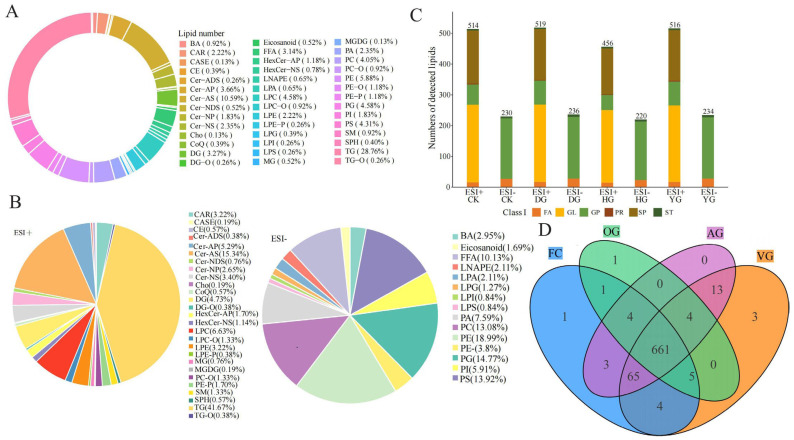
Lipid identification of *C. sinensis*. (**A**) Lipid classification of *C. sinensis*; (**B**) lipid pie charts in ESI+ and ESI− modes; (**C**) number of lipids detected in positive and negative ion modes between different groups; (**D**) Venn plots of samples from 4 groups, FC, VG, AG, and OG.

In the 4 groups of samples, a total of 765 lipid compounds were identified (Table S2) and categorized into 42 subclasses (Figure 1A), with the 5 most abundant classes being TG (*n* = 220, 28.76%), Cer-AS (*n* = 81, 10.59%), PE (*n* = 45, 5.88%), LPC (*n* = 35, 4.58%), and PG (*n* = 35. 4.58%).

Due to the varying ionization tendencies of lipids, a total of 528 lipids classes were detected in ESI+ mode, including TG (41.67%), Cer-AS (15.34%), and LPC (6.63%). In ESI− mode, 237 lipids classes were identified, with PE (18.99%), PG (14.77%), and PS (13.92%) being the most prevalent (Figure 1B). Additionally, the numbers of lipids identified in ESI+ mode were 514 for FC, 519 for VG, 456 for OG, and 516 for AG. In ESI− mode, the counts for FC, VG, OG, and AG were 230, 236, 220, and 234, respectively (Figure 1C). Notably, the number of GLs, GPs, and SPs constituted a significant proportion of the total lipid count. The Venn plot results indicated that the total number of lipids identified across FC, VG, OG, and AG amounted to 661 (Figure 1D). Interestingly, the FC group exhibited the highest lipid count, followed by the VG group. Except for the AG group, FC, VG, and OG groups displayed specific lipid profiles. In summary, while there was considerable overlap in the lipid compositions between fresh *C. sinensis* (FC) and those subjected to various drying methods, the lipidomic profiles revealed notable differences.

### 3.2. Multivariate Statistical Analysis of Lipids in C. sinensis Under Different Drying Methods

**Figure 2:**
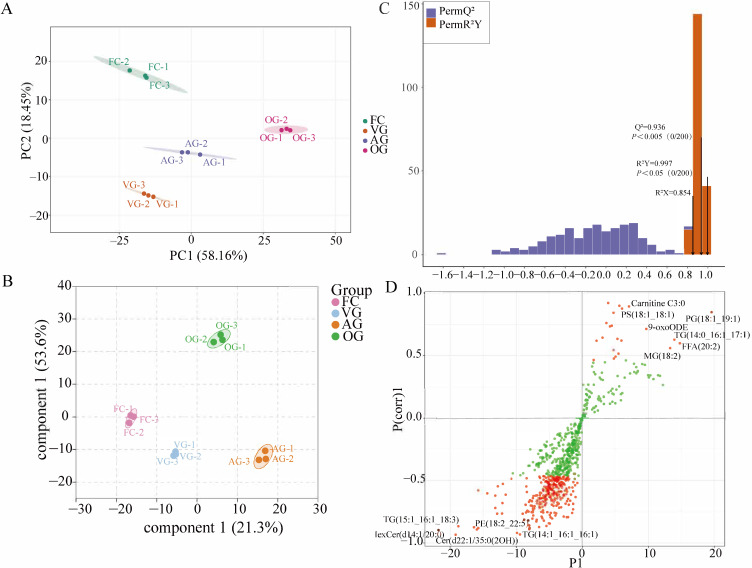
(**A**) PCA diagram. The first principal component’s variability is represented by PC1, while PC2 corresponds to the variability of the second principal component. Different samples are depicted as dots, and groups are illustrated on the right side. (**B**) OPLS-DA analysis diagram. The horizontal and vertical axes reflect the extent to which components are interpreted, with various samples represented by dots and distinct groups shown on the right side. (**C**) OPLS-DA validation diagram. The horizontal axis indicates the R^2^Y and Q^2^ values of the model, while the vertical axis displays the frequency at which the model’s classification effect appears across 200 random permutation tests. In the diagram, the orange color denotes the R^2^Y values from the random permutation model, the purple color illustrates the Q^2^ values from the same model, and the black arrows signify the original model’s R^2^X, R^2^Y, and Q^2^ values. (**D**) S-plot of OPLS-DA.

By graphically representing the primary two principal component (PC) scores derived from the variables categorized within the FC, VG, OG, and AG groups of data, we can visually compare the differences between samples subjected to various drying methods. The scores were plotted separately using PC1 (56.16%) and PC2 (18.45%), as illustrated in Figure 2A, resulting in an identification rate of 74.61%. Notably, three biological replicates from each of the four groups clustered closely together, thereby affirming the reliability and consistency of the LC-MS/MS technique. It is important to note that PCA has limitations regarding its ability to eliminate random errors and inter-group variations while maintaining data integrity.

In contrast, OPLS-DA serves as a supervised statistical method for discriminant analysis, providing a more comprehensive approach to identifying differences between groups [41]. The OPLS-DA model was utilized to identify differential abundance lipids (DALs) associated with various drying methods. In the score plot of OPLS-DA (Figure 2B), the distinct populations of *C. sinensis* across different drying techniques were consistent with the findings from the PCA. The model’s fitting quality and validity were further supported by 200 random permutation tests, yielding R^2^X = 0.854, R^2^Y = 0.997, and Q^2^ = 0.936. These values indicate that the model exhibits strong fitting performance and predictive capability (Figure 2C). The results from the S-Plot revealed that the various drying methods were associated with a diverse array of lipids. Notably, the lipids located closer to the lower left and right corners of the plot indicated more significant differences between the drying methods. Specific lipid compounds that demonstrated substantial differences included TG(14:1_16:1_16:1), TG(17:1_18:1_20:1), TG(15:1_16:1_18:3), Cer(d22:1/35:0(2OH)), HexCer(d14:1/20:0), PE(18:2_22:5), PG(18:1_19:1), FFA(20:2), MG(18:2), TG(14:0_16:1_17:1), 9-oxoODE, and PS(18:1_18:1) (Figure 2D).

**Figure 3:**
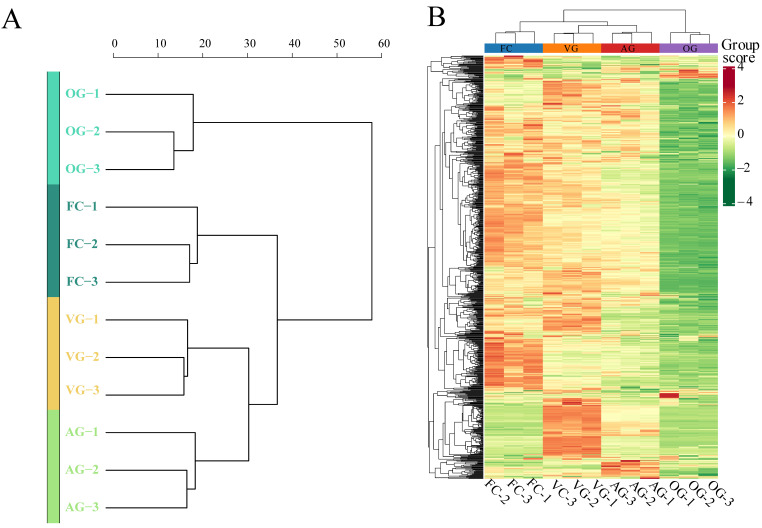
(**A**) Sample hierarchical clustering tree. Each branch in the graph represents a sample. Samples with high similarity are clustered in the same cluster. (**B**) Clustered heatmaps. The name of the sample is shown horizontally, and the right side represents the different groups.

Cluster analysis employing a hierarchical approach was performed on samples from various comparison groups to construct a cluster tree and a cluster heatmap that illustrate the similarity among the samples, as presented in Figure 3A,B. Samples that clustered together exhibited a higher degree of similarity. Notably, the four sample groups were generally categorized into two main categories: the FC, VG, and AG groups formed one category, while the OG group constituted a separate category. In addition, further analysis revealed that the VG and AG groups could be classified together into a distinct subgroup.

### 3.3. Screening of Different Accumulated Lipids (DALs)

**Figure 4:**
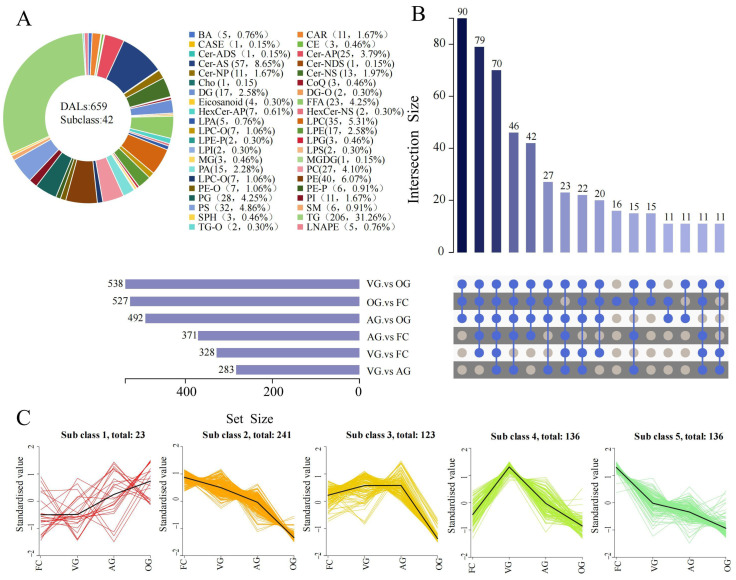
Examination of DALs in *C. sinensis* subjected to various drying methods. (**A**) Pie chart illustrating all subclasses of DALs. (**B**) Venn diagrams showcasing DALs across different comparison categories. (**C**) K-means clustering analysis of all DALs, where the horizontal axis denotes sample groupings, and the vertical axis represents standardized relative lipid content.

The results obtained from OPLS-DA indicate that the VIP values generated through multivariate analysis facilitate the categorical identification of differential lipid accumulation among the samples. Additionally, the *p*-values derived from univariate analyses can be integrated to further identify differentially accumulated lipids. The differential accumulation lipids (DALs) investigated in this research were determined based on the criteria of VIP > 1 and *p* < 0.05. A comprehensive list of 659 DALs annotated by *C. sinensis* across various drying techniques is presented in Table S3. Furthermore, 42 lipid subclass variables (such as TG, Cer-AS, PE, LPC, PC, etc.) significantly influenced the distinctions among the four sample groups, as illustrated in Figure 4A. K-means cluster analysis was employed to elucidate the variation in these 659 DALs across different drying methods. Based on their accumulation patterns, these DALs could be categorized into five subclasses, as shown in Figure 4C. This illustrates the variety and intricacy of lipids in *C. sinensis* subjected to various drying techniques. The alterations in the lipids of *C. sinensis* were more significant when employing the OG and AG methods compared to those dried through the VG method.

**Figure 5:**
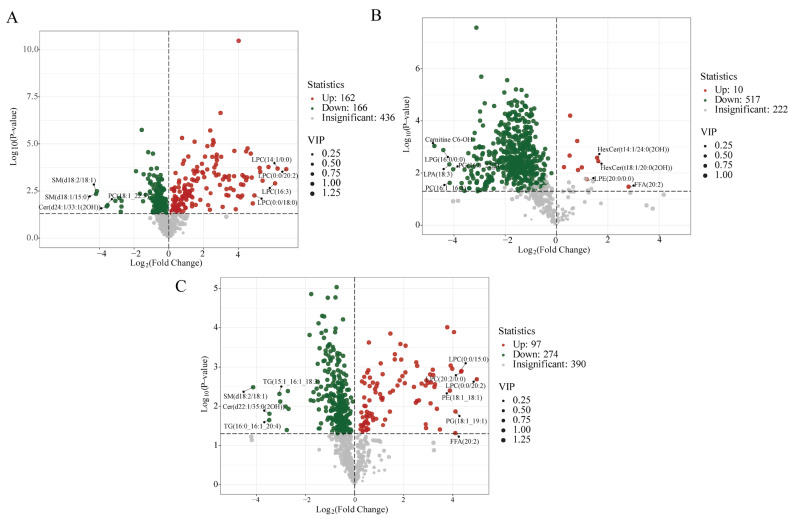
Volcano grams of comparative groups of *C. sinensis*. (**A**) VG vs. FC; (**B**) AG vs. FC; (**C**) OG vs. FC.

Variations in the expression levels of metabolites among samples subjected to various drying techniques, along with their statistical significance, are illustrated through volcano plots (Figure 5). Each point on the volcano plot corresponds to a specific metabolite.

A greater absolute value on the x-axis signifies a higher FC in expression between the samples. Conversely, a larger value on the y-axis indicates a more significant differential expression, thereby enhancing the reliability of the identified metabolites. Changes in differential abundance levels (DALs) between VG and FC are presented in Figure 5A, where DALs were significantly up-regulated for LPC (14:1/0:0), LPC (0:0/20:2), LPC (18:3/0:0), and LPC (0:0/18:0). In contrast, DALs for SM (d18:2/18:1), SM (d18:1/15:0), Cer (d24:1/33:1 (2OH)), and PC (18:1_22:5) were significantly down-regulated. LPC plays an important role in inflammation associated with cardiovascular and neurodegenerative diseases in humans. The expression of various classes of LPC was significantly up-regulated under cold stress conditions. As shown in Figure 5B, the lipid categories of AG vs. FC (including FFA (20:2), PG (18:1_19:1), PE (18:1_18:1), and LPC (0:0/20:2)) in the comparison group exhibited significant up-regulation. In contrast, TG (16:0_16:1_20:4), Cer (d22:1/35:0 (2OH)), SM (d18:2/18:1), and TG (15:1_16:1_18:3) showed significant down-regulation. Changes in DALs between OG and FC are depicted in Figure 4C, where most lipids were down-regulated, particularly Carnitine C6-OH, LPG (16:0/0:0), LPA (18:3), and PC (16:1_16:1). Conversely, GP, SP, and FA analogs, such as FFA (20:2), LPE (20:0/0:0), HexCer (t18:1/20:0(2OH)), and HexCer (t14:1/24:0(2OH)), were the most significantly up-regulated metabolites. Given the changes in lipid composition, it is plausible that the TG in *C. sinensis* undergoes degradation and oxidation during the drying process, which may reduce both its nutritional value and biological activity.

### 3.4. Identification of Key DALs

**Figure 6:**
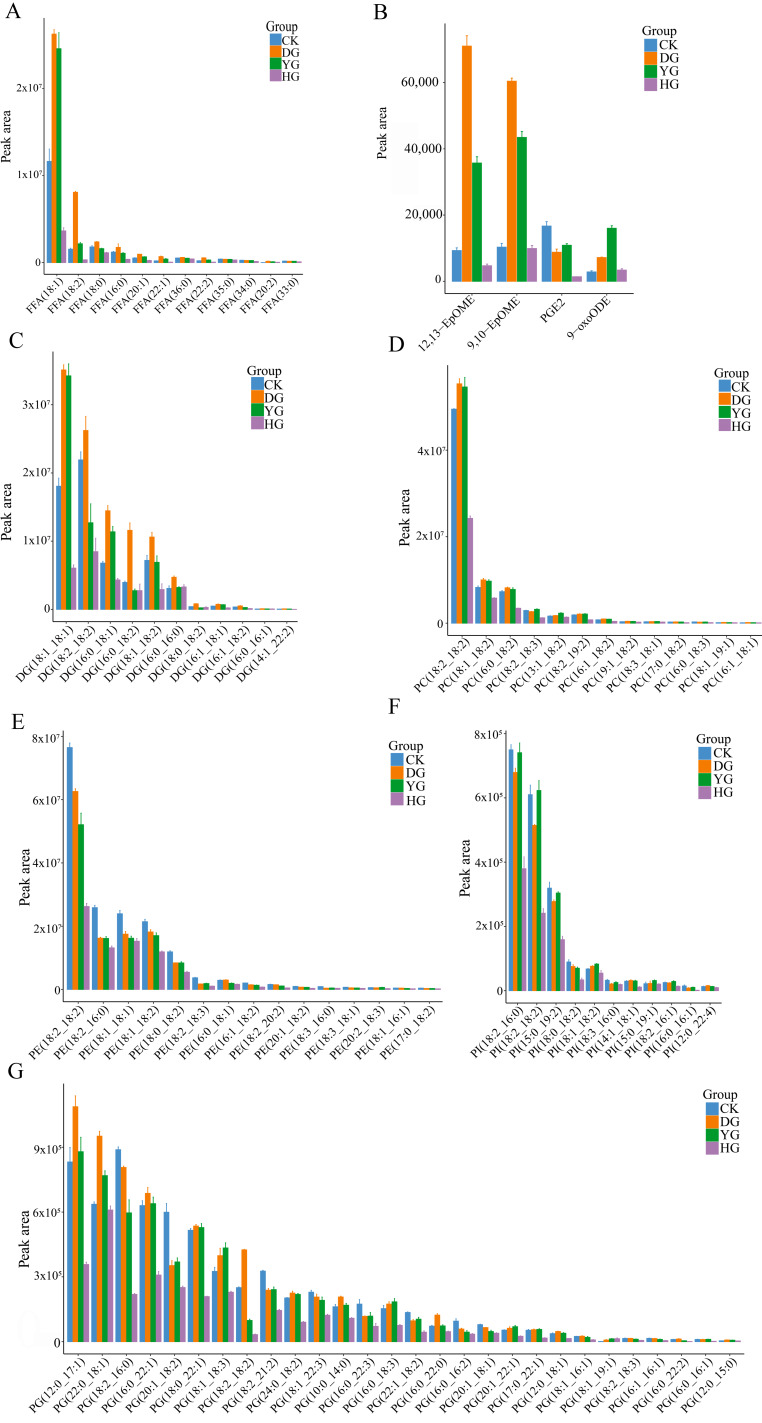
Peak areas of bioactive: (**A**) FFAs; (**B**) eicosanoids; (**C**) DGs; (**D**–**G**) GPs (PC, PE, PI, and PG) detected in *C. sinensis* under fresh and studied drying methods.

This study focused on various bioactive lipids found in DALs. The peak regions of the bioactive lipids observed in *C. sinensis* using various drying techniques are clearly illustrated in Figure 6. The area of the peaks corresponds to the quantity of each lipid present in the sample. When examining the peak areas across the four groups of FFAs (Figure 6A), it was noted that only two compounds, FFA (18:1) and FFA (18:2), exhibited significant differences among the groups. Eicosanoid, a biologically active oxidized lipid formed through oxidation, is also analyzed [42]. A comparison of the peak areas of eicosanoid in the four groups revealed significant differences, with the VG and AG groups displaying higher peak areas (Figure 6B). In addition, when comparing the peak areas of the four DG groups (Figure 6C), the peak area content of the VG group was higher than that of the other group. Upon comparing the peak areas of the GPs (PC, PE, PI, and PG) across the four groups, it was observed that the compounds exhibited greater diversity, with all groups showing significant differences from one another, particularly demonstrating higher peak areas in the FC and VG groups.

### 3.5. Functional Annotation and Enrichment Analysis of DALs

**Figure 7:**
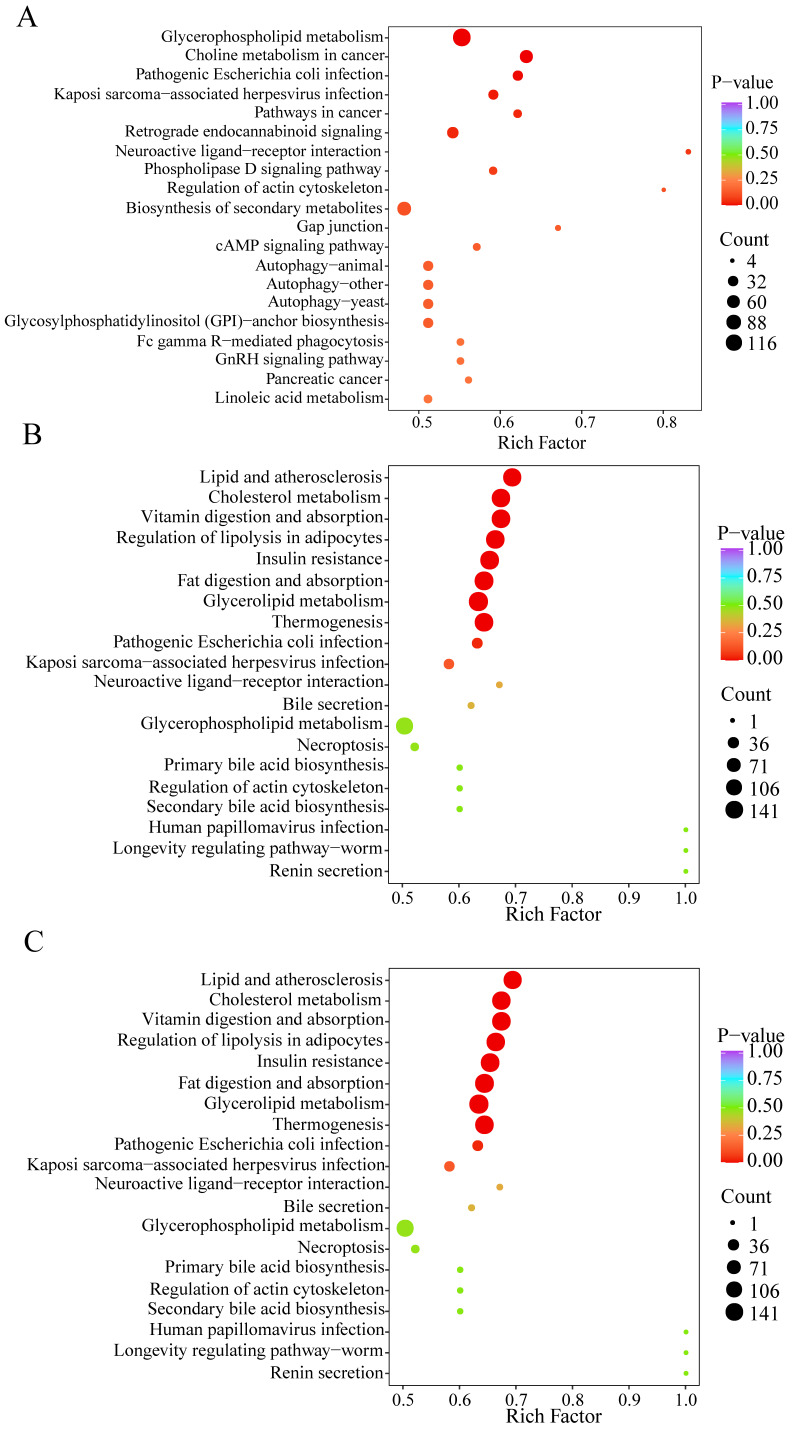
Differential lipid pathway enrichment map. (**A**) VG vs. FC; (**B**) AG vs. FC; (**C**) OG vs. FC. The horizontal coordinate indicates the corresponding rich factor of each pathway, the vertical coordinate is the pathway name (sorted by *p*-value), and the color of the dots reflects the *p*-value size, and the redder the color, the more significant the enrichment.

To further investigate lipid metabolism in *C. sinensis* subjected to various drying methods, analyses of metabolic pathways were performed on differential lipids associated with function in the FC group compared to the VG, AG, and OG groups. Using criteria for significantly related pathways (*p* < 0.1), *p*-values and pathway impacts were evaluated to identify pathways with significant enrichment through pathway topology analysis. The distinct lipids identified are primarily involved in 15 metabolic pathways (Figure 7), including glycerophospholipid metabolism, lipid metabolism, glycerolipid metabolism, etc. Among the comparison groups, six co-enriched pathways were shared, namely glycerophospholipid metabolism, neuroactive ligand–receptor interaction, and Kaposi’s sarcoma-associated herpesvirus infection.

### 3.6. Interpretation of Critical Pathways

**Figure 8:**
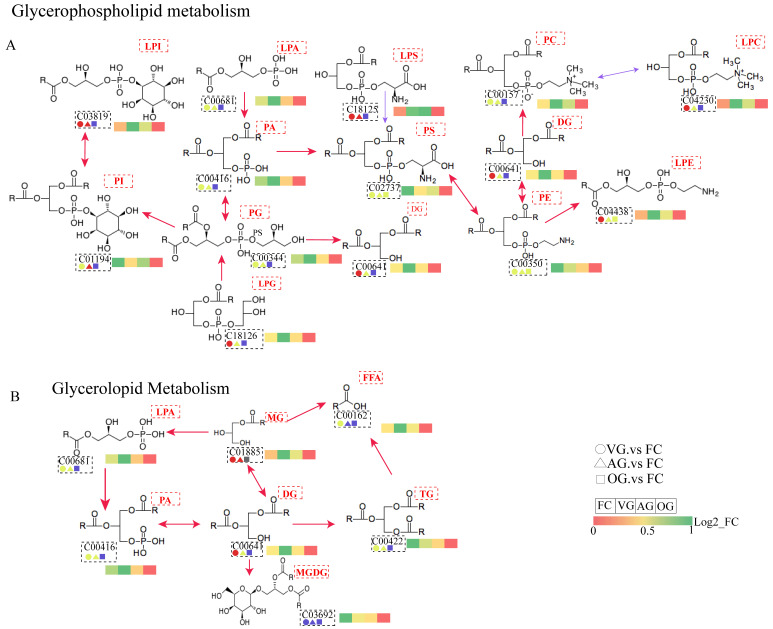
The metabolic pathways of major lipids in *C. sinensis* under inter-group comparisons, the numbers represent the ID of each lipid subclass: (**A**) glycerophospholipid metabolism; (**B**) glycerolipid metabolism. Note: red indicates that the lipid content is significantly up-regulated, blue indicates that the lipid content is significantly down-regulated, gray indicates that the lipid was detected but not significantly changed, and yellow indicates that both up-regulated and down-regulated lipids were included.

To further analyze the lipid metabolism of *C. sinensis* following drying, key metabolic pathways were examined in the FC group compared to the VG, AG, and OG groups (Figure 8). Based on the criteria of significantly related pathways (*p* < 0.1), the metabolic pathways involving lipids exhibited differences in *C. sinensis* dried using the three distinct methods. Metabolic mapping based on key differential lipids indicated that PC, PE, PS, PI, TG, and PA, among others, were primarily involved in glycerophospholipid metabolism, as illustrated in Figure 8A. Glycerophospholipids, the most abundant class of phospholipids in the body, participate in protein recognition and membrane signaling, thereby playing a crucial role in cellular function [40]. 

Phosphatidylcholine (PC) and phosphatidylethanolamine (PE) act as substrates for the synthesis of phosphatidylserine (PS), a process facilitated by phosphatidylserine synthase (PTDSS), which mediates the base exchange between these substrates and serine. This mechanism results in a temporary accumulation of PC and PE, contributing to extensive lipid oxidation reactions [43]. Analysis revealed the down-regulation of lipid subclass compounds in the OG versus FC glycerophospholipid metabolic pathway.

Metabolic mapping based on key differential lipids indicated that LPA, FFA, MG, VG, PA, TG, and MGVG were primarily involved in glycerolipid metabolism, as illustrated in Figure 8B. Analysis revealed that lipid subclasses within the glycerolipid metabolic pathway were down-regulated in both the OG versus FC comparison groups. Diacylglycerol (VG) has the potential to prevent increases in lipid concentration in the liver, thereby mitigating obesity, cardiovascular diseases, and various other metabolic disorders from their root causes [44]. Notably, VG analogs were found to be up-regulated in the VG vs. FC comparison group.

## 4. Discussion

Metabolomics primarily focuses on hydrophilic categories, while lipidomics is considered a separate group due to the complexity of the biological lipidome [45]. The various drying methods have distinct impacts on the lipids extracts of *C. sinensis*, which may be linked to their mechanisms of action in higher organisms.

The hot-air-drying (OG) technique, which promotes the transfer of moisture from the interior of the material to its surface using heated air, is often favored for its low financial investment. However, the elevated temperature of 60 °C can accelerate oxidation, resulting in a decline in quality and a reduction in the nutrients available in *C. sinensis* [46]. The VG method operates by creating a pressure differential between atmospheric pressure and a vacuum, effectively reducing the levels of oxygen and humidity in the surrounding environment. This pressure change is crucial to the drying process, as it not only facilitates moisture removal but also helps preserve the integrity of the materials being processed. The successful application of VG spans various edible products and other materials, demonstrating its versatility and effectiveness in maintaining quality.

When comparing VG to conventional methods, such as OG, the advantages of using VG become evident. Materials dried using VG tend to retain superior quality compared to those treated with OG. This is likely due to the enhanced preservation of flavor, texture, and nutritional content resulting from reduced exposure to oxygen and moisture during the drying process [47,48]. Consequently, VG represents a promising approach in the field of drying technologies, particularly for those involved in food preservation and material processing. The VG method is widely regarded as the most effective drying technique for preserving quality, primarily because it mitigates two crucial factors: oxygen exposure and elevated temperatures [49]. Indeed, under the VG method, we noted that lipid species, along with changes in *C. sinensis*, experienced lesser impacts compared to alternative drying approaches.

This observation likely arises from the rapid freezing of the material, followed by heating in a vacuum environment, which enhances lipid stability while markedly decreasing the rates of hydrolysis and oxidation associated with the lipids. The VG method is commonly acknowledged as the most efficient drying technique for maintaining quality, largely due to its ability to reduce two vital elements: exposure to oxygen and high temperatures. In fact, the up- and down-regulated lipids were more stable in the VG method compared to the FC comparator group, and, thus, the VG method had less effect on the modification of Chinese saponins compared to other drying techniques. This phenomenon is probably a result of the swift freezing of the material, succeeded by heating in a vacuum setting, which improves lipid stability while significantly lowering the hydrolysis and oxidation rates linked to the lipids [50].

A lipidomic approach was employed to identify 765 lipid metabolites from *C. sinensis*, encompassing glycerophospholipids, glycerides, and sphingolipids. Similar findings have been reported in related studies [41]. A higher proportion of glycerophospholipids is associated with enhanced functional activity and the comparatively strong *C. sinensis*. It has been documented that glycerophospholipids may contribute to the prevention of intestinal cancer while also reducing the risk of obesity and cardiovascular diseases [51,52]. The different lipids identified were mainly involved in 15 metabolic pathways (Figure 7), of which glycerophospholipid metabolism and lipid metabolism showed significant enrichment. Lipid metabolism refers to the process of synthesis, degradation, and transport of lipids by the organism [53]. As shown in Figure 8, metabolic mapping based on significantly different lipids—PE, LPE, PA, DG, PG, PI, PC, LPC, and P—is mainly involved in glycerophospholipid and lipid metabolism.

Glycerophospholipids, as a kind of lipid molecule, can be involved in the synthesis and degradation of lipids through metabolic pathways [54]. However, drying significantly diminished the content of various glycerophospholipids, with the OG method exhibiting the greatest reduction in lipid content. This decrease may be attributed to enzymatic lipid degradation or lipid transformation during high-temperature drying [55]. Previous research has indicated that elevated temperatures during the drying process accelerate the oxidation of lipids, the oxidation of proteins, and the degradation of phospholipids in edible mushrooms [56]. Additionally, other studies have found that interconversion occurs between phospholipids and between triglycerides (TG) and phospholipids [57]. Glycerophospholipid represents the primary lipid category found in cellular membranes and is crucial for various cellular functions [58]. Changes in glycerophospholipid levels due to drying may affect the fluidity and permeability of these membranes, potentially resulting in lipid leakage and ultimately leading to the degradation and oxidation of lipids [59].

Lipids play a crucial role in numerous cellular processes, particularly fatty acids (such as unsaturated free fatty acids and eicosanoids) and glycerophospholipids (including phosphatidylglycerol, phosphatidylcholine, phosphatidylethanolamine, and phosphatidylinositol). Free fatty acids (FFAs) are an important source of energy for human tissues and possess physiological functions, including the promotion of insulin secretion and anti-inflammatory effects [60]. Eicosanoids are believed to mediate their signaling effects only in the form of free acids, which can regulate renal, cardiovascular, and gastrointestinal functions. PG, PC, PE, and PI, as the four major GPs, play significant roles in promoting cell and tissue growth, maintaining metabolism, and enhancing the body’s immunity and regenerative capacity [61,62].

The reduction in phospholipids may lead to the production of FFA and oxidation products. The triglycerides (TG) present in *C. sinensis* are characterized by a high ratio of long-chain fatty acids and an increased level of unsaturation. Additionally, there is a notable rise in the content of free fatty acids (FFA). Triglycerides (TG) are formed through the process of lipid hydrolysis. Notably, when comparing the YG and HG groups with the CK group, a significant reduction in TG levels was observed, leading us to hypothesize that a considerable degree of hydrolysis occurred in *C. sinensis* under these two drying techniques. Within living organisms, triglycerides are broken down by lipase into free fatty acids and glycerol. Subsequently, these components can enter glucose metabolism or be incorporated into other metabolic pathways following a series of enzyme-mediated reactions. The increase in free fatty acids (FFAs) could result from the breakdown of macromolecular lipids, and it is conceivable that these free fatty acids influence the flavor profile of *C. sinensis*, a hypothesis that warrants further investigation. Lysophosphatidylcholine (LPC) is increasingly recognized as a key factor positively associated with cardiovascular and neurodegenerative diseases and as a major phospholipid component of oxidized low-density lipoprotein (Ox-LDL) [63]. In the VG. vs. FC comparison group, LPC content was significantly up-regulated. In the OG. vs. FC comparison group, LPC content was significantly down-regulated. This may be because cold storage reduces lipid degradation [64].

In an untargeted metabolomics study, lipid and lipid-like molecules were the main metabolites of *C. sinensis* and the main differential accumulation of metabolites (DAMs) of *C. sinensis* under different drying methods, and the content and class of lipid and lipid-like molecules were correlated with the antioxidant capacity of *C. sinensis* [17]. This shows that the quality of *C. sinensis* is significantly affected under different drying methods. Therefore, in this study, the lipid composition and content of *C. sinensis* were analyzed in detail under different drying methods. In short, variations in the levels of oxidation-related lipids among the three drying methods may be linked to their distinct drying mechanisms and differing lipid metabolic processes. The use of the lipidomic approach is an important way to elucidate the lipid content and composition of *C. sinensis* during drying.

## 5. Conclusions

In this study, we conducted a comparative analysis of the effects of different drying methods—namely, VG, OG, and AG—on the lipid extracts derived from *C. sinensis*, while also presumably the potential mechanisms involved in lipid oxidation through lipidomics. Our findings revealed that the predominant lipid classes present in *C. sinensis* include glycerophospholipids, glycerides, and sphingolipids, with glycerophospholipids comprising nearly 50% of the total lipid content. We identified significant differences in lipid metabolites among the studied groups, with 328, 371, and 527 differential cumulative lipid metabolites detected in the FC (control) versus VG, AG, and OG groups, respectively. The analysis indicated that among the drying methods, VG had the least impact on lipid content, whereas OG exerted the most pronounced effect. These variances in detected lipid classes can be linked to processes such as degradation, transformation, and oxidation of lipids induced by the drying methods employed. Furthermore, our KEGG pathway analyses highlighted three metabolic pathways that exhibited commonality across the groups: glycerophospholipid metabolism, linoleic acid metabolism, and triglyceride metabolism. These pathways are likely associated with lipid oxidation that occurs during the drying process of *C. sinensis*. The insights gained from this study may serve as a valuable reference for selecting optimal drying methods and for further research aimed at enhancing the quality of lipid extracts derived from *C. sinensis*.

## Data Availability

The original contributions presented in the study are included in the article/Supplementary Materials, further inquiries can be directed to the corresponding author.

## Supplementary Materials

The following supporting information can be downloaded at: https://www.mdpi.com/article/10.3390/jof10120855/s1, Table S1: complete information about internal; Table S2: detailed information of 765 lipids detected by *Cordyceps sinensis* under different drying methods; Table S3: detailed information of 659 lipids with significant differences was detected in *Cordyceps sinensis* under different drying methods. Figure S1: (A) total ion chromatograms of NEG, (B) total ion chromatograms of POS, Figure S2: the coefficient of variation (CV) diagram of quality control samples.
