# Supplementary figures and images for: Evaluation of Lipid Changes During the Drying Process of Cordyceps sinensis by Ultra Performance Liquid Chromatography–Tandem Mass Spectrometry (UPLC-MS/MS)-Based Lipidomics Technique
Source: J Fungi (Basel). 2024 Dec 11;10(12):855. doi: 10.3390/jof10120855 (PMC11677378; doi:10.3390/jof10120855)

A

NEG

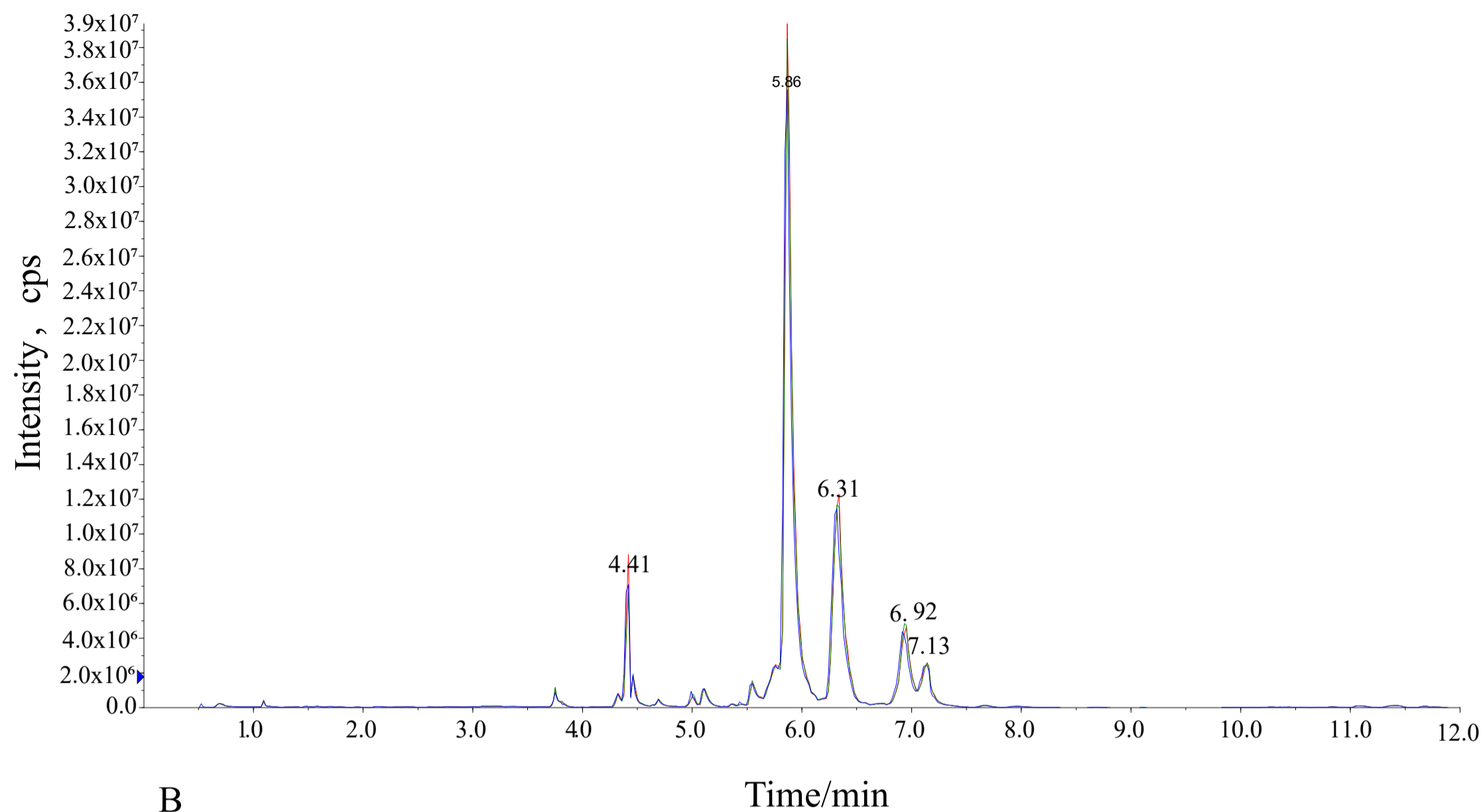

B

POS

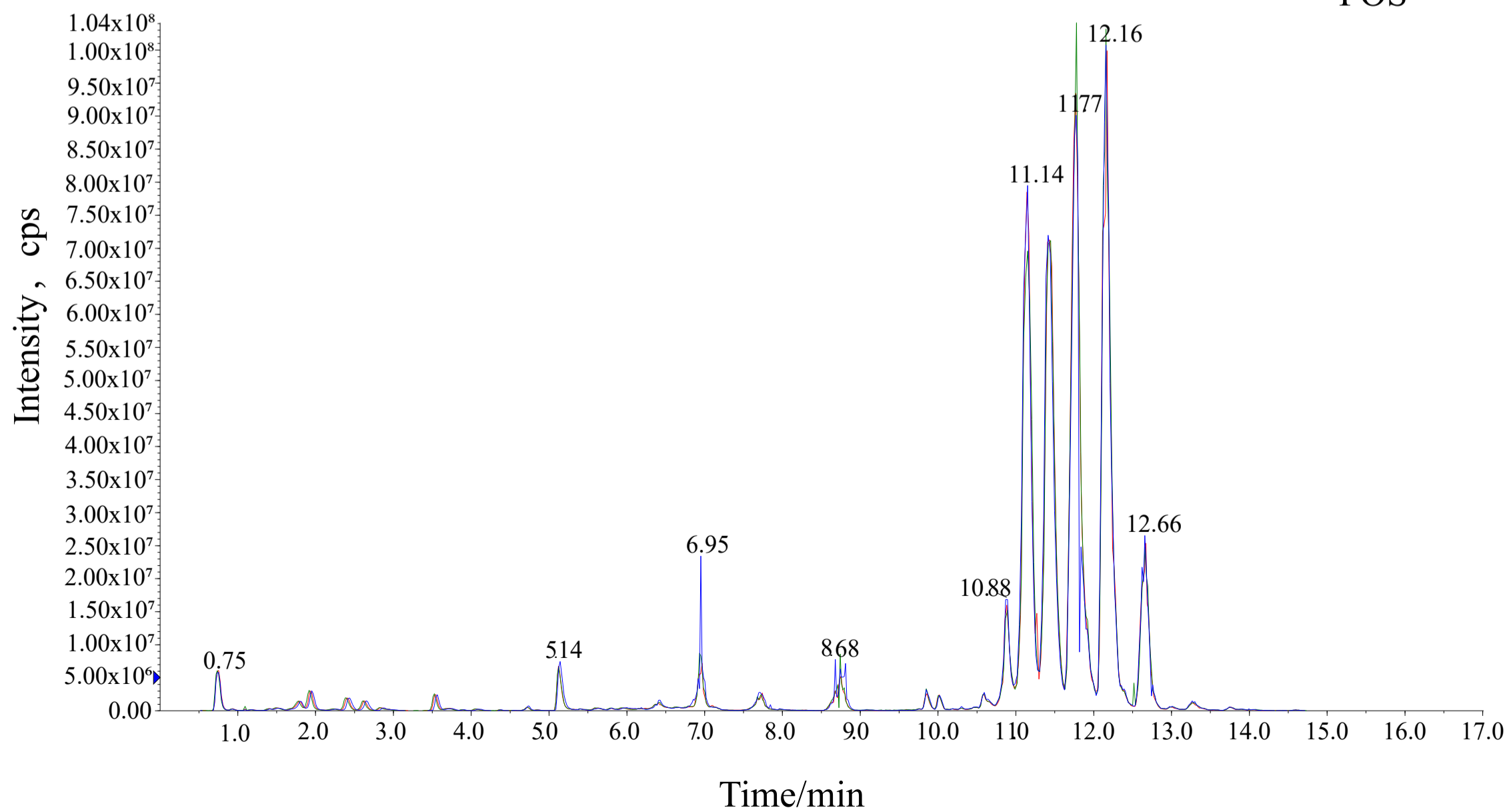

Supplement: Supplementary file 1 [file jof-10-00855-s001.zip › Figure.S1.pdf]

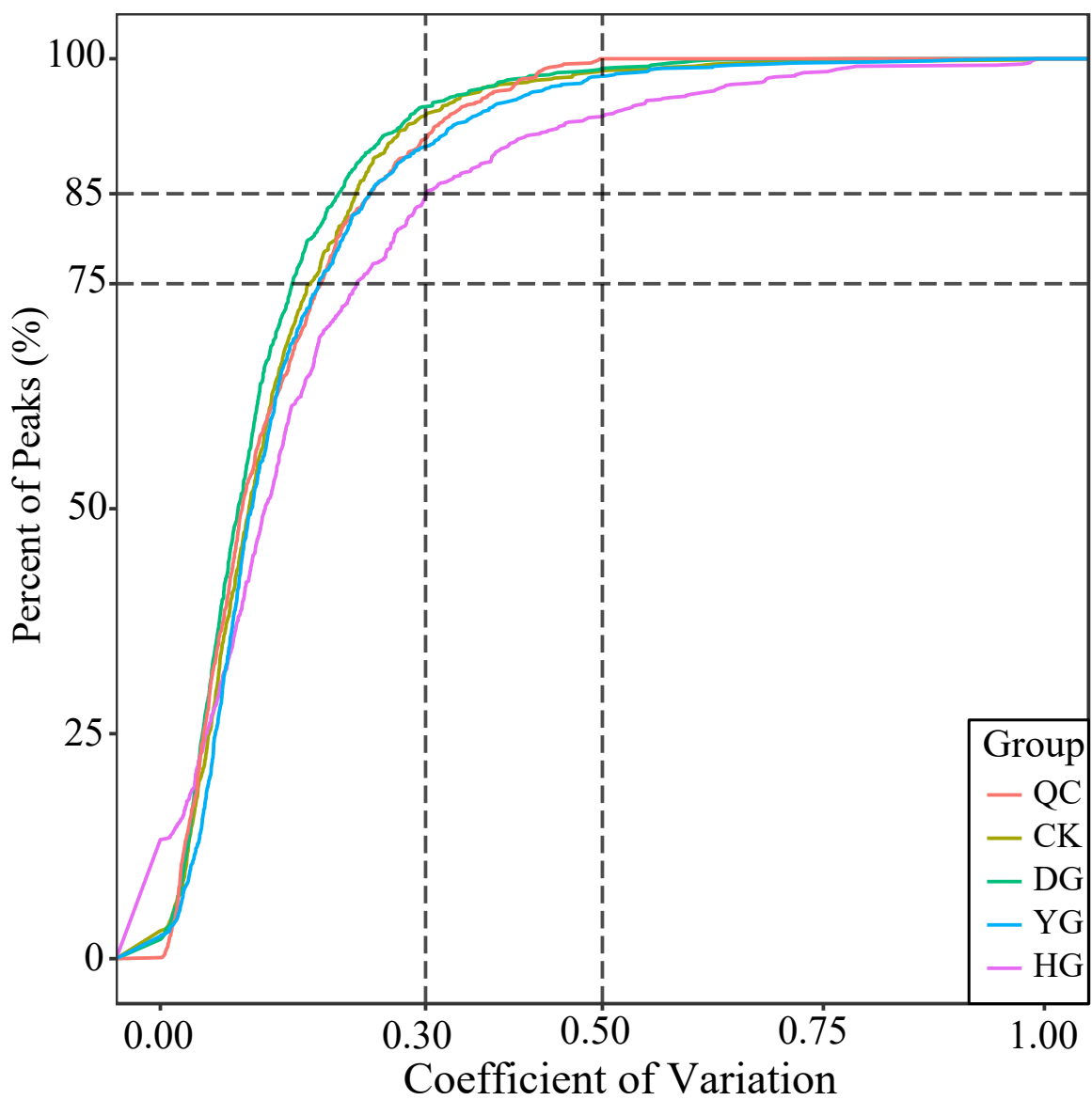

Supplement: Supplementary file 1 [file jof-10-00855-s001.zip › Figure.S2.pdf]
